# Cost-effective production of alginate oligosaccharides from *Laminaria japonica* roots by *Pseudoalteromonas agarivorans* A3

**DOI:** 10.1186/s12934-023-02170-7

**Published:** 2023-09-09

**Authors:** Xiao-Hui Sun, Xiu-Lan Chen, Xiao-Fei Wang, Xin-Ru Zhang, Xiao-Meng Sun, Mei-Ling Sun, Xi-Ying Zhang, Yu-Zhong Zhang, Yu-Qiang Zhang, Fei Xu

**Affiliations:** 1 State Key Laboratory of Microbial Technology, Marine Biotechnology Research Center, Shandong University, Qingdao, 266237 China; 2 Frontiers Science Center for Deep Ocean Multispheres and Earth System & College of Marine Life Sciences, Ocean University of China, Qingdao, 266003 China; 3 Laboratory for Marine Biology and Biotechnology, Pilot National Laboratory for Marine Science and Technology, Qingdao, 266237 China

**Keywords:** Alginate oligosaccharides, *Pseudoalteromonas*, *Laminaria japonica*, Fermentation, Alginate lyase

## Abstract

**Background:**

Alginate oligosaccharides (AOs) are the degradation products of alginate, a natural polysaccharide abundant in brown algae. AOs generated by enzymatic hydrolysis have diverse bioactivities and show broad application potentials. AOs production via enzymolysis is now generally with sodium alginate as the raw material, which is chemically extracted from brown algae. In contrast, AOs production by direct degradation of brown algae is more advantageous on account of its cost reduction and is more eco-friendly. However, there have been only a few attempts reported in AOs production from direct degradation of brown algae.

**Results:**

In this study, an efficient *Laminaria japonica*-decomposing strain *Pseudoalteromonas agarivorans* A3 was screened. Based on the secretome and mass spectrum analyses, strain A3 showed the potential as a cell factory for AOs production by secreting alginate lyases to directly degrade *L. japonica*. By using the *L. japonica* roots, which are normally discarded in the food industry, as the raw material for both fermentation and enzymatic hydrolysis, AOs were produced by the fermentation broth supernatant of strain A3 after optimization of the alginate lyase production and hydrolysis parameters. The generated AOs mainly ranged from dimers to tetramers, among which trimers and tetramers were predominant. The degradation efficiency of the roots reached 54.58%, the AOs production was 33.11%, and the AOs purity was 85.03%.

**Conclusion:**

An efficient, cost-effective and green process for AOs production directly from the underutilized *L. japonica* roots by using strain A3 was set up, which differed from the reported processes in terms of the substrate and strain used for fermentation and the AOs composition. This study provides a promising platform for scalable production of AOs, which may have application potentials in industry and agriculture.

**Supplementary Information:**

The online version contains supplementary material available at 10.1186/s12934-023-02170-7.

## Background

Brown algae, including *Laminaria japonica*, *Laminaria digitata*, *Undaria pinnatifida*, *Macrocystis pyrifera*, *Ascophyllum nodosum*, *Sargassum natans*, and others, are a large group of marine seaweeds (~ 1800 species) with huge biomass [1, 2]. They have aroused a wide interest by virtue of their rapid growth, superior nutritional value and absence of recalcitrant lignin [3, 4]. Among brown algae, *L. japonica* is an important primary producer in the coastal ocean and is cultivated as a popular edible alga worldwide, especially in East Asia [5]. In food processing, while *L. japonica* blade is always in demand, its so-called “root”, which includes meristem and stipe, is usually discarded in the food industry, causing a waste of resources. Thus, techniques for the high-value use of *L. japonica* roots are worth exploring.

Alginate is an acid polysaccharide, consisting of mannuronic acid (M) and its epimer guluronic acid (G) linked via 1,4-*O*-glycosidic bonds. It is abundant in various brown algae, accounting for 30–60% of the dry weight [6], and thus is a significant marine carbon source. Alginate is frequently employed as a thickener, stabilizer, and gel-forming agent [7]. It is also the raw material for the production of alginate oligosaccharides (AOs).

AOs are the depolymerization products of alginate with low degrees of polymerization (DPs), which have recently received widespread attention because of their diverse bioactivities. As reported, AOs have various physiological activities, including antitumor, antioxidant, prebiotic activities, and others [8–10], and thus have broad therapeutic potentials. In agriculture, AOs can promote the root growth, improve the tolerance to drought and induce the immunity of plants [11–13]. In particular, AOs at DP2-4 have been shown to have a variety of bioactivities on plants [14], for instance, promoting the root development in *Triticum aestivum* L [15]. and increasing the yield of flowering Chinese cabbage [16]. The conventional method of AOs production is to hydrolyze alginate by heating in the presence of acid, such as hydrochloric acid, sulfuric acid and formic acid [17]. Acid hydrolysis is a rapid method with violent chemical reaction, leading to the directly break of glycosidic bonds between the C1 and C4 in alginate [18]. The use of acids and the requirement for high temperatures result in environmental pollution and energy waste [19]. By comparison, AOs production via enzymolysis using alginate lyases is milder, more environment-friendly and energy-saving as no acids or intense heating is required. Moreover, AOs derived from enzymatic degradation are unsaturated oligomers with lower DPs, which have stable structures and more biological activities [18]. As reported, unsaturated AOs prepared by enzymolysis have excellent antioxidant activity and better immunomodulatory effect on murine macrophage RAW264.7 cells compared with AOs prepared by acid hydrolysis [20, 21]. Thus, the enzymatic approach is an advantageous method for AOs preparation.

Alginate lyases, a class of polysaccharide lyases (PLs) distributed in 14 families in the Carbohydrate-Active enZYmes Database (http://www.cazy.org/), cleave the 1,4-glycosidic bonds between alginate monomers via the β-elimination reaction, leading to the formation of an unsaturated double bond at the non-reducing end [22]. So far, the conventional enzymatic approach for AOs production is to use alginate lyases to degrade sodium alginate. However, due to the multiple steps required to extract sodium alginate from brown algae [23], the high cost of using sodium alginate as substrate for the industrial AOs preparation is a non-negligible issue. Accordingly, producing AOs by directly degrading brown algae is more advantageous on account of its cost reduction. Till now, only 3 studies have reported the direct production of AOs from brown algae. Li et al. provided an approach for preparing AOs directly from brown seaweed via a combination of enzymatic hydrolysis and selective fermentation by utilizing an engineered *Yarrowia lipolytica* strain [24]. Wang et al. isolated an alginate lyase-producing *Bacillus litoralis* strain that can degrade *Sargassum horneri* to produce AOs [25]. Additionally, the alginate lyase AlgL7 from *Microbulbifer* sp. ALW1 that was heterologously expressed in *Escherichia coli*, was capable of producing AOs from *L. japonica* [26]. Therefore, more approaches for preparing AOs directly from brown seaweeds, which are simple, economic and efficient, need to be developed.

In this study, we screened a strain *Pseudoalteromonas agarivorans* A3 from *L. japonica* collected from a kelp farm in Weihai, China. Strain A3 can directly decompose *L. japonica* mainly relying on its secreted alginate lyases. We optimized the fermentation conditions for alginate lyase production of strain A3 and the hydrolysis parameters of the fermentation broth supernatant (FBS) on *L. japonica* roots. Moreover, we characterized the degradation products of *L. japonica* roots by the FBS. Based on our data, an efficient, cost-effective and green process for AOs production directly from *L. japonica* roots by utilizing the FBS of strain A3 was set up, providing a new enzymatic method for AOs production.

## Results and discussion

### Screening of an effective *L. japonica*-decomposing bacterial strain

**Fig. 1:**
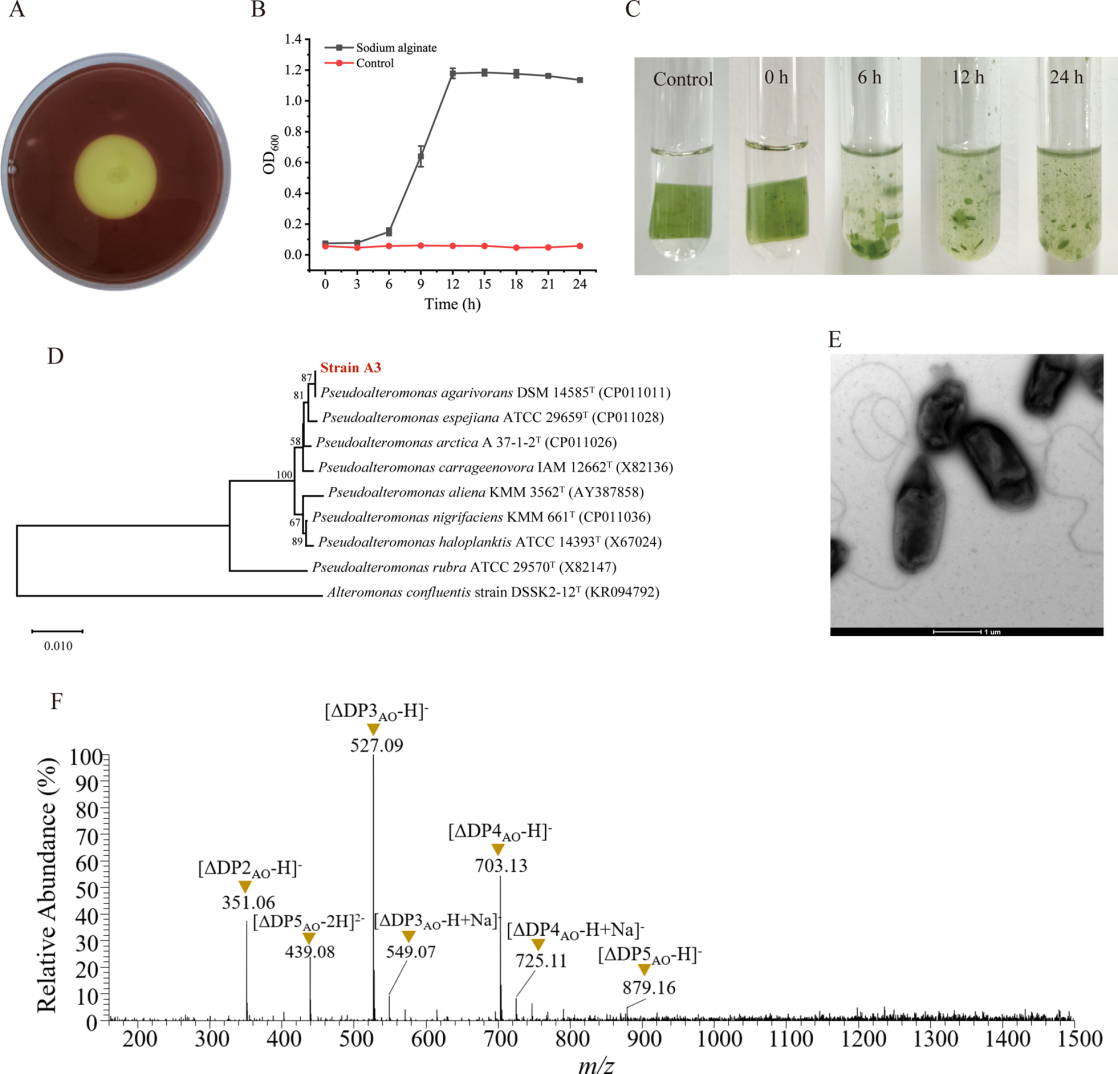
Screening of the *L. japonica*-decomposing bacterial strain *Pseudoalteromonas agarivorans* A3. **A** the degradation of alginate by strain A3. Strain A3 was cultured on the screening plate at 25 °C for 5 days and the plate were dyed with Lugol’s solution to determine the degradation of alginate around the strain colony. **B** the growth curve of strain A3 cultured in the liquid medium containing 0.5% sodium alginate as the sole carbon source. The growth curve of strain A3 cultured in the liquid medium without sodium alginate was taken as the control. **C** decomposition of *L. japonica* by strain A3. Strain A3 was cultured at 25 °C in the medium containing a piece of *L*. *japonica* blade and 3% sea salts. The decomposition of *L*. *japonica* was observed at 0, 6, 12 and 24 h. The *L*. *japonica* blade-containing medium without strain A3 incubated at 25 °C, 180 rpm for 24 h was taken as the control. **D** the neighbor-joining phylogenetic tree of strain A3 based on the 16S rRNA gene sequences. The bootstrap values of each branch were tested by 1000 repetitions. **E** transmission electron micrograph of negatively stained cells of strain A3 cultured in 2216E marine broth at 25 °C for 12 h. **F** LTQ-Orbitrap-MS analysis of the degradation products of the *L*. *japonica* piece by the fermentation broth supernatant of strain A3. *DP* degree of polymerization, *AO* alginate oligosaccharide

To obtain bacteria with high *L. japonica-*decomposing ability, bacterial strains with alginate-hydrolyzing ability were first isolated from the *L*. *japonica* sample by detecting the clear halo around the bacterial colony on the screening plates containing alginate as the sole carbon source. Then, to further verify the utilization of alginate, 5 strains with a high ratio of the halo diameter (HD) to the colony diameter (CD) (the HD/CD ratio ≧ 3) were cultured in the liquid medium with sodium alginate as the sole carbon source. Among them, strain A3 showed the highest HD/CD ratio (5.3, Fig. 1A) and the fastest growth in the liquid medium, reaching the stationary phase after 12 h (Fig. 1B). When cultured in the medium containing a piece of *L. japonica* blade and 3% (w/v) sea salts, strain A3 showed the highest *L. japonica-*decomposing ability. After 12 h-cultivation, the *L*. *japonica* piece was completely degraded into debris (Fig. 1C), indicating that strain A3 is an effective *L. japonica*-decomposing strain. Thus, strain A3 was chosen for further study and its genome was sequenced.

Phylogenetic analysis based on 16S rRNA gene sequences revealed that strain A3 was affiliated with the *Pseudoalteromonas* genus (Fig. 1D), sharing the highest 16S rRNA gene sequence similarity (99.93%) with that of *Pseudoalteromonas agarivorans* DSM 14585, a strain capable of decomposing algal polysaccharides such as agar, alginate and carrageenan [27]. Transmission electron microscopy (TEM) analysis showed that cells of strain A3 were rod-shaped with a single polar flagellum, approximately 1.6–2.4 μm in length and 0.8–1 μm in width (Fig. 1E), which resembles the cell morphology of *P. agarivorans* DSM 14585 [27]. Therefore, strain A3 is a novel strain of *Pseudoalteromonas agarivorans*, designated as *Pseudoalteromonas agarivorans* A3.

Several brown alga-decomposing bacterial strains have been reported. *Geobacillus thermodenitrificans* OS27 degraded the brown alga *Undaria pinnatifida* into small pieces after 5 days [28] and *Microbulbifer* sp. 6532 A after 2 days [29]. *Pseudoalteromonas* sp. Alg6B showed obvious hydrolysis of *L. japonica*, transforming *L. japonica* pieces into particles after 24 h [30]. Comparatively, strain A3 showed a stronger *L. japonica*-decomposing ability, suggesting that strain A3 may have a potential in *L. japonica* degradation in industry.

### Ability of strain A3 to produce AOs directly from *L. japonica* via its secreted alginate lyases

Represented by alginate, various polysaccharides are the major components of *L. japonica*. In the genome of strain A3, a number of genes encoding putative carbohydrate-active enzymes (CAZymes) were observed, including dozens of genes encoding enzymes responsible for the degradation of algae-specific polysaccharides (e.g. fucoidan, laminarin and alginate) and other plant polysaccharides (e.g. cellulose and starch). To investigate the enzymes secreted by strain A3 involved in the *L. japonica* decomposition, we analyzed the secretome of strain A3 cultured with *L. japonica* as the sole carbon source. In the secretome, 9 CAZymes were detected, including 4 glycoside hydrolases (GHs) and 5 PLs (Table 1). The 4 GHs belong to the GH13 and GH16 families. Thereinto, the 2 GH13 enzymes (OIZ54_07165 and OIZ54_18130) share the highest sequence similarities with an α-amylase (GenBank: P29957.3) and a pullulanase (GenBank: P07206.2) [31, 32], respectively, indicating that they may be involved in the degradation of the starch in *L. japonica*. A putative GH16 laminarinase (OIZ54_02445) was detected, which may hydrolyze the abundant laminarin in *L. japonica*. Another detected GH16 enzyme (OIZ54_00850) shares the highest sequence similarity with an agarase based on the amino acid sequence. Remarkably, all of the 5 putative alginate lyases of strain A3 were detected in the secretome, which are from the PL6, -7, -17, and -18 families (Table 1). Among them, the PL18 alginate lyase was the most abundant (48.90%), and the 5 alginate lyases had a total abundance of 70.88% (Table 1). These results suggested that the alginate lyases secreted by strain A3 played a crucial role in the *L. japonica* decomposition.

**Table 1:** Secretome analysis of the extracellular carbohydrate-active enzymes of strain A3 cultured with *L. japonica* blade as the sole carbon source

Locus tag	Family	PSMs	Putative function^a^	Abundance^b^ (%)
OIZ54_07305	PL18	89	Alginate lyase	48.90
OIZ54_07165	GH13	18	α-amylase	9.89
OIZ54_09065	PL6	16	Alginate lyase	8.79
OIZ54_09060	PL17	11	Alginate lyase	6.04
OIZ54_09025	PL6	8	Alginate lyase	4.40
OIZ54_00850	GH16	8	β-agarase	4.40
OIZ54_02445	GH16	7	Laminarinase	3.85
OIZ54_18130	GH13	6	Pullulanase	3.30
OIZ54_12210	PL7	5	Alginate lyase	2.75

^a^Putative function was predicted by blasting the protein sequence against the NCBI database

^b^Abundance was calculated based on the proportion of the PSMs of an enzyme in the sum of PSMs of all carbohydrate-active enzymes in the secretome. The enzymes with the abundance less than 2% in the secretome are not listed

To investigate whether strain A3 can decompose *L. japonica* with the secreted enzymes to produce AOs, we utilized the FBS of strain A3 cultured with *L. japonica* to degrade *L. japonica* blade and analyzed the degradation products by LTQ-Orbitrap-MS. The main peaks all correspond to the molecular masses of unsaturated AOs (including dimers, trimers, tetramers and pentamers) (Fig. 1F). The degradation products of starch and laminarin, namely glucose monomers or oligomers, were not observed, which may be due to the low contents of starch and laminarin in *L. japonica* and the small amount of starch- and laminarin-degrading enzymes in the FBS of strain A3. These results showed that the products released from *L. japonica* by the FBS of strain A3 were mainly AOs. Thus, strain A3 may have a potential in AOs production from brown algae in industry.

### Optimization of the alginate lyase production of strain A3 by single factor and response surface optimization (RSM) methods

**Fig. 2:**
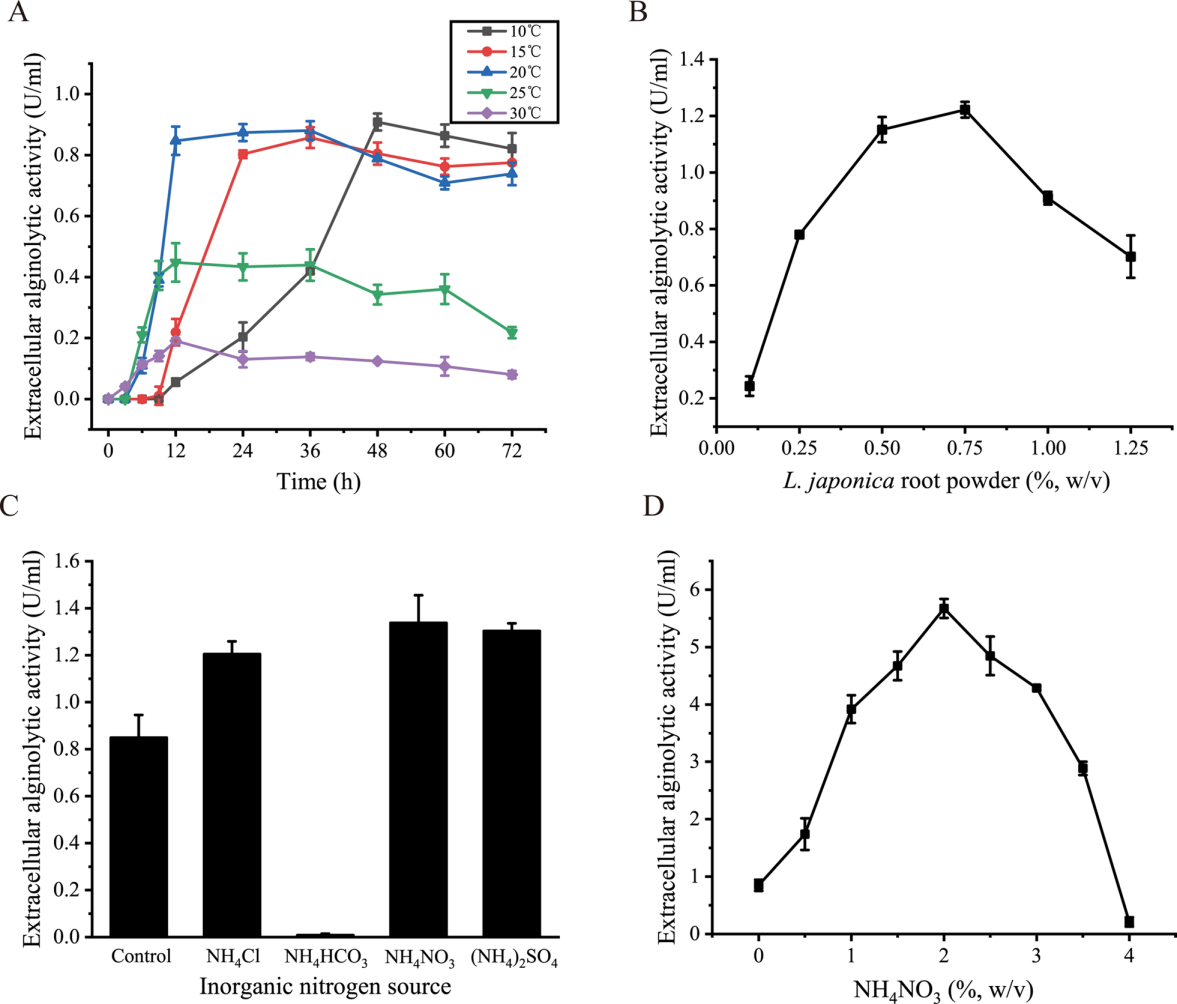
Optimization of the fermentation conditions of strain A3 for alginate lyase production by single factor method. **A** effects of fermentation temperature and time on the alginate lyase production. Strain A3 was cultured at 10, 15, 20, 25 or 30 °C in the basic fermentation medium. **B** effect of the content of *L. japonica* root powder on the alginate lyase production. Strain A3 was cultured in the medium containing 3% sea salts and different concentrations of *L. japonica* root powder (0.1, 0.25, 0.5, 0.75, 1 or 1.25%) at 20 °C for 24 h. **C** effects of inorganic nitrogen sources on the alginate lyase production. Strain A3 was cultured in the basic fermentation medium with an addition of 0.13% (w/v) NH_4_Cl, NH_4_HCO_3_, NH_4_NO_3_ or (NH_4_)_2_SO_4_ at 20 °C for 24 h. The alginate lyase production of strain A3 cultured in the basic fermentation medium at 20 °C for 24 h was taken as the control. **D** effect of the concentration of NH_4_NO_3_ on the alginate lyase production. Strain A3 was cultured in the basic fermentation medium containing different concentrations of NH_4_NO_3_ (0, 0.5, 1.0, 1.5, 2.0, 2.5, 3.0, 3.5 or 4%) at 20 °C for 24 h

Single factor assays were first performed to optimize the alginate lyase production of strain A3. The effects of fermentation temperature and time, *L. japonica* root powder content, and inorganic nitrogen sources on the alginate lyase production of strain A3 were investigated. The highest alginate lyase production of strain A3 cultured at 10 °C, 15 °C and 20 °C were almost identical, higher than that at 25 °C or 30 °C, and strain A3 reached the highest alginate lyase production the fastest (~ 12 h) at 20 °C (Fig. 2A). Thus, 20 °C and 12 h were chosen as the optimum fermentation temperature and time, respectively. In addition, for the alginate lyase production of strain A3, the optimum content of *L. japonica* root powder was 0.75% (Fig. 2B). Among the 4 inorganic nitrogen sources, NH_4_Cl, NH_4_NO_3_ and (NH_4_)_2_SO_4_ had promoting effects on the alginate lyase production of strain A3, and NH_4_NO_3_ was the optimal one (Fig. 2C). In contrast, the growth of strain A3 was seriously inhibited by NH_4_HCO_3_ with unknown reasons, resulting in a significant decrease in the alginate lyase production (Fig. 2C). After optimization, the optimal concentration of NH_4_NO_3_ for the alginate lyase production was 2% (Fig. 2D).

Based on the single factor experimental results, the fermentation parameters were further optimized by RSM, including the Plackett–Burman (PB) design, the steepest ascent (descent) experiment and the central composite design (CCD). In the PB design, 8 variables (NH_4_NO_3_ concentration, rotation speed, *L. japonica* root powder content, fermentation time, fermentation temperature, pH, inoculum size, and sea salt concentration) were investigated to determine the significant factors affecting the alginate lyase production of strain A3 (Table 2). Based on the analysis of variance (ANOVA) of the PB design results, the *p*-values of NH_4_NO_3_ concentration, fermentation temperature, pH and inoculum size were less than 0.05 (Additional file 1: Table S1), indicating that these 4 variables were the significant factors affecting the alginate lyase production and were selected for further optimization by performing the steepest ascent (descent) experiment [33]. The steepest ascent (descent) experiment started from the center point of the 4 key variables in the PB design and moved along the path, in which NH_4_NO_3_ concentration, pH and inoculum size increased, while fermentation temperature decreased. The highest response (8.56 U/ml) was reached at the experiment with 2% NH_4_NO_3_, 19.5 °C, pH 8.0 and 1.75% inoculum size, indicating that this point was closed to the maximum response region of alginate lyase production (Table 3). CCD was used to determine the optimal level of each key variable and the effect of their interactions for the alginate lyase production of strain A3. Thirty experiments were performed (Table 4), and the equation was established by multiple regression analysis (Additional file 1: Formula S1).

**Table 2:** Plackett–Burman (PB) design for screening of culture conditions affecting the alginate lyase production of strain A3

Run	*X*_1_	*X*_2_	*X*_3_	*X*_4_	*X*_5_	*X*_6_	*X*_7_	*X*_8_	*X*_9_	*X*_10_	*X*_11_	Enzyme activity (U/ml)
1	1.2	180	0.4	36	25	7	2	3	1	− 1	− 1	1.10
2	1.2	120	0.6	24	25	8	1	3	1	1	− 1	1.51
3	1.2	120	0.4	36	20	8	2	2	1	1	1	4.37
4	1.8	180	0.6	24	20	7	2	2	1	1	− 1	4.11
5	1.8	120	0.4	24	25	7	2	3	− 1	1	1	1.39
6	1.8	180	0.4	36	25	8	1	2	− 1	1	− 1	2.02
7	1.8	120	0.6	36	25	7	1	2	1	− 1	1	1.26
8	1.2	180	0.6	24	25	8	2	2	− 1	− 1	1	2.24
9	1.2	180	0.6	36	20	7	1	3	− 1	1	1	3.59
10	1.8	180	0.4	24	20	8	1	3	1	− 1	1	4.42
11	1.2	120	0.4	24	20	7	1	2	− 1	− 1	− 1	2.46
12	1.8	120	0.6	36	20	8	2	3	− 1	− 1	− 1	6.67

*X*_1_, NH_4_NO_3_ (w/v, %); *X*_2_, rotation speed (rpm); *X*_3_, *L. japonica* root powder (w/v, %); *X*_4_, fermentation time (h); *X*_5_, fermentation temperature (°C); *X*_6_, pH; *X*_7_, inoculum size (%); *X*_8_, sea salt concentration (w/v, %); *X*_9_, *X*_10_ and *X*_11_, dummy variables

**Table 3:** Experiment design of steepest ascent (descent) and corresponding response

Experiment number	NH_4_NO_3_ (%)	Fermentation temperature (°C)	pH	Inoculum size (%)	Enzyme activity (U/ml)
1	1.5	22.5	7.5	1.25	2.36
2	1.75	21	7.75	1.5	4.42
3	2	19.5	8.0	1.75	8.56
4	2.25	18	8.25	2	8.28
5	2.5	16.5	8.5	2.25	0.16

**Table 4:** Experiment design of central composite design (CCD) experiment and corresponding response

Run	*X*_1_		*X*_2_		*X*_3_		*X*_4_		Enzyme activity (U/ml)
	Coded level	Real level (%)	Coded level	Real level (^o^C)	Coded level	Real level	Coded level	Real level (%)	
1	− 1	1.75	− 1	18.5	− 1	7.85	− 1	1.5	6.53
2	1	2.25	− 1	18.5	− 1	7.85	− 1	1.5	7.40
3	− 1	1.75	1	20.5	− 1	7.85	− 1	1.5	5.08
4	1	2.25	1	20.5	− 1	7.85	− 1	1.5	6.23
5	− 1	1.75	− 1	18.5	1	8.15	− 1	1.5	7.04
6	1	2.25	− 1	18.5	1	8.15	− 1	1.5	7.44
7	− 1	1.75	1	20.5	1	8.15	− 1	1.5	6.1
8	1	2.25	1	20.5	1	8.15	− 1	1.5	6.93
9	− 1	1.75	− 1	18.5	− 1	7.85	1	2	6.86
10	1	2.25	− 1	18.5	− 1	7.85	1	2	7.26
11	− 1	1.75	1	20.5	− 1	7.85	1	2	6.75
12	1	2.25	1	20.5	− 1	7.85	1	2	6.85
13	− 1	1.75	− 1	18.5	1	7.85	1	2	7.48
14	1	2.25	− 1	18.5	1	7.85	1	2	7.64
15	− 1	1.75	1	20.5	1	7.85	1	2	7.47
16	1	2.25	1	20.5	1	7.85	1	2	8.01
17	− 2	1.5	0	19.5	0	8	0	1.75	5.69
18	2	2.5	0	19.5	0	8	0	1.75	6.35
19	0	2	− 2	17.5	0	8	0	1.75	7.46
20	0	2	2	21.5	0	8	0	1.75	6.49
21	0	2	0	19.5	− 2	7.7	0	1.75	5.34
22	0	2	0	19.5	2	8.3	0	1.75	6.21
23	0	2	0	19.5	0	8	− 2	1.25	6.80
24	0	2	0	19.5	0	8	2	2.25	8.60
26	0	2	0	19.5	0	8	0	1.75	8.52
26	0	2	0	19.5	0	8	0	1.75	8.34
27	0	2	0	19.5	0	8	0	1.75	8.24
28	0	2	0	19.5	0	8	0	1.75	7.91
29	0	2	0	19.5	0	8	0	1.75	7.94
30	0	2	0	19.5	0	8	0	1.75	8.31

*X*_1_, NH_4_NO_3_ (w/v, %); *X*_2_, fermentation temperature (°C); *X*_3_, pH; *X*_4_, inoculum size (%)

**Fig. 3:**
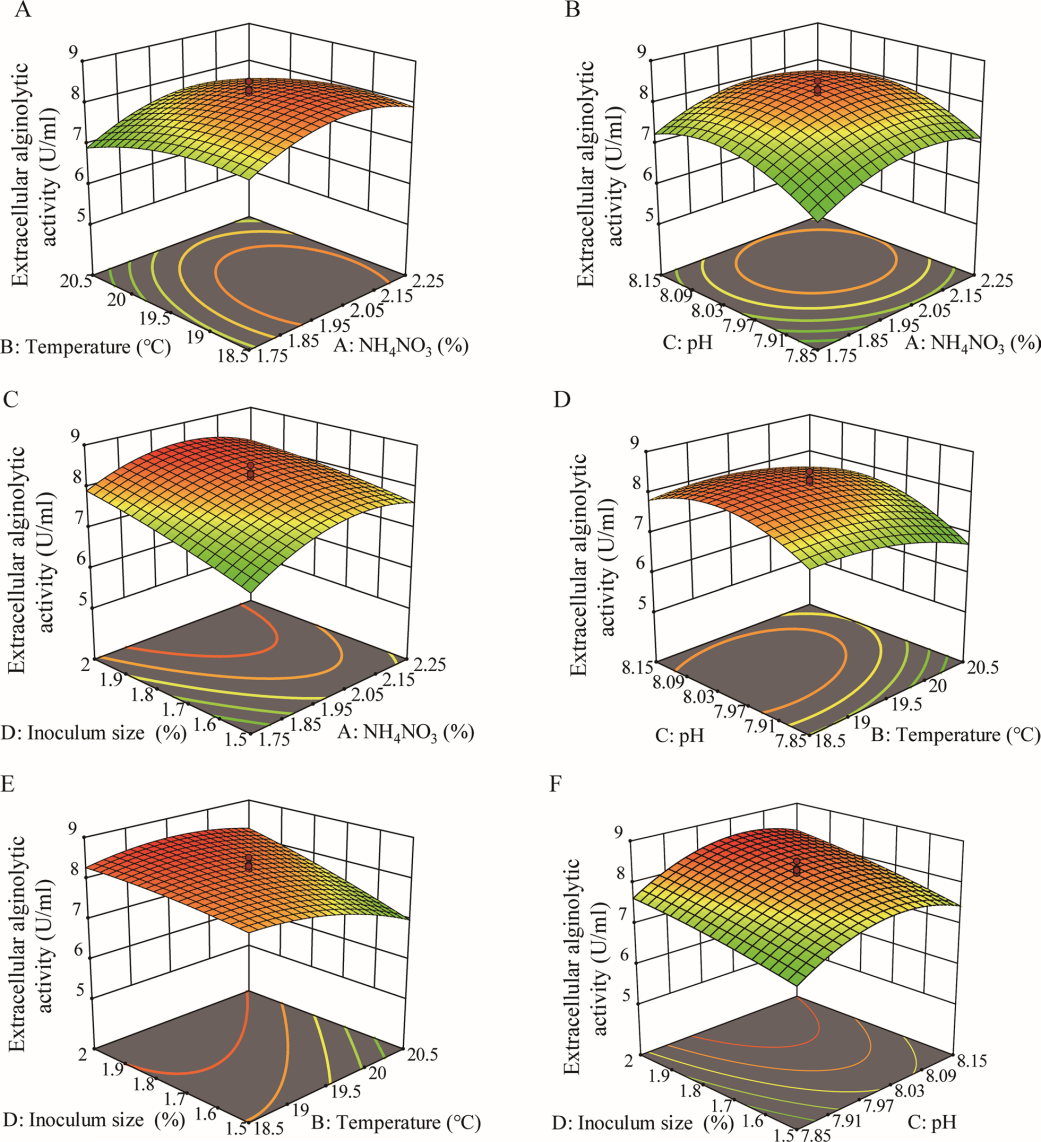
Three-dimensional plots of the effect of four variables on the alginate lyase production of strain A3. **A** interaction of fermentation temperature and NH_4_NO_3_ concentration. **B** Interaction of pH and NH_4_NO_3_ concentration. **C** Interaction of inoculum size and NH_4_NO_3_ concentration. **D** Interaction of pH and fermentation temperature. **E** Interaction of inoculum size and fermentation temperature. **F** Interaction of inoculum size and pH. When the effect of 2 variables was plotted, the other variables were set at the central level

**Fig. 4:**
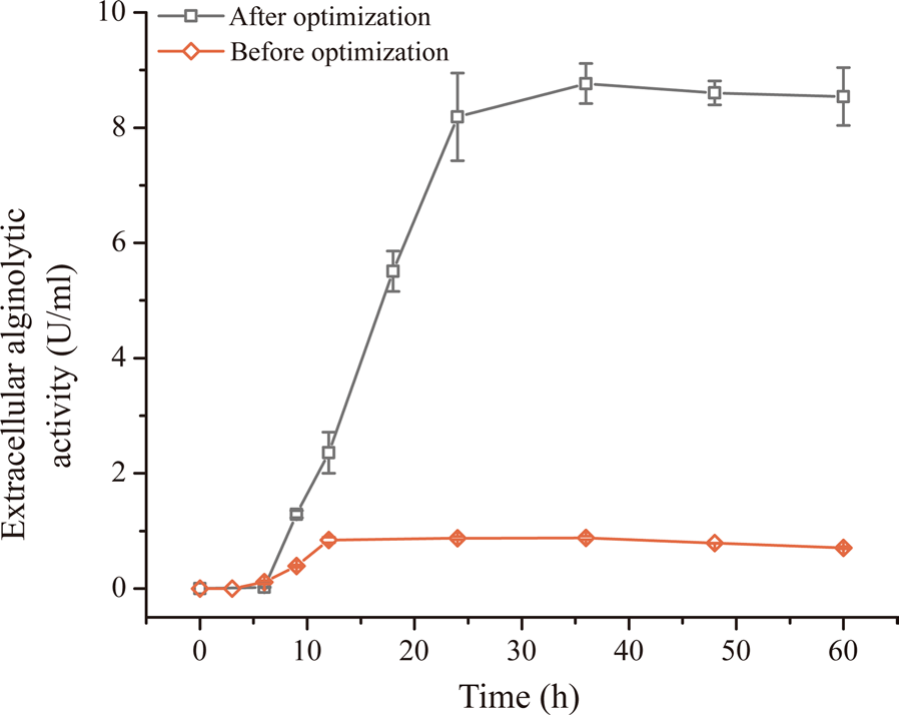
The alginate lyase production of strain A3 cultured under the conditions before and after optimization. Before optimization, strain A3 was cultured in the medium (pH 8.0) containing 1% (w/v) *L. japonica* root powder and 3% sea salts with 1% inoculum size at 20 °C, 180 rpm for 24 h. After optimization, strain A3 was cultured in the medium (pH 8.04) containing 2.03% NH_4_NO_3_, 0.6% *L. japonica* root powder and 3% sea salt with 2% inoculum size at 19.6^o^C, 120 rpm for 36 h. The graphs show data from triplicate experiments (mean ± S.D.)

The ANOVA results showed that the determination coefficient *R*^*2*^, Model *F*-value, Lack of Fit *p*-value of the model were 0.9527, 21.57, and 0.3244, respectively, and the *p*-value of the model was less than 0.0001, suggesting that the regression equation had nice fitting result and this model had a high significance (Additional file 1: Table S2). As shown in Fig. 3, except for the contour map of response surface between NH_4_NO_3_ concentration and pH, those between other variables were elliptical or saddle, demonstrating that the interactions between the corresponding variables were significant. Based on the analysis using Design Expert 12 software, the maximum alginate lyase production was predicted to be 8.57 U/ml under the optimum conditions (2.03% NH_4_NO_3_, 0.6% *L. japonica* root powder, 3% sea salt, 2% inoculum size, pH 8.04, 19.56 °C and 120 rpm for 36 h). To validate the feasibility of the model, the fermentation of strain A3 was performed under the predicted optimum conditions. The alginate lyase production reached the maximum 8.77 ± 0.16 U/ml (649.76 ± 8.28 U/ml detected by the ultraviolet absorption (UA) method), approximately 10.4 times higher than that before optimization (0.84 ± 0.097 U/ml), indicating a good accordance with the prediction production and an effective optimization (Fig. 4).

Up to now, many alginate-degrading strains have been reported. Wang et al. isolated a strain *Bacillus litoralis* M3 that could secrete alginate lyases to degrade alginate. When strain M3 was cultured in the medium containing sodium alginate, the enzyme activity of the FBS was 0.4079 U/ml (the UA method) [25]. Li et al. screened an alginate-degrading strain *Pseudoalteromonas* sp. SM0524. The alginolytic activity of its fermentation broth reached 62.61 U/ml (the UA method) [34]. In addition, Zhu et al. screened 26 alginate-metabolizing strains, and the strain *Microbulbifer* sp. ALW1 showed the highest extracellular alginate lyase activity, reaching 0.18 U/ml (the DNS method) [35]. The alginate lyase production of strain A3 was much higher than that of the above strains, indicating that the FBS of strain A3 was a promising enzyme cocktail for the AOs preparation. Moreover, after optimization, the fermentation medium only contains NH_4_NO_3_, seawater, and *L. japonica* roots that are underutilized in the food industries, which is simple, accessible and low-cost, providing feasibility for industrial production of the FBS of strain A3.

### Optimization of the hydrolysis parameters of the FBS of strain A3 for AOs preparation from *L. japonica* roots

**Fig. 5:**
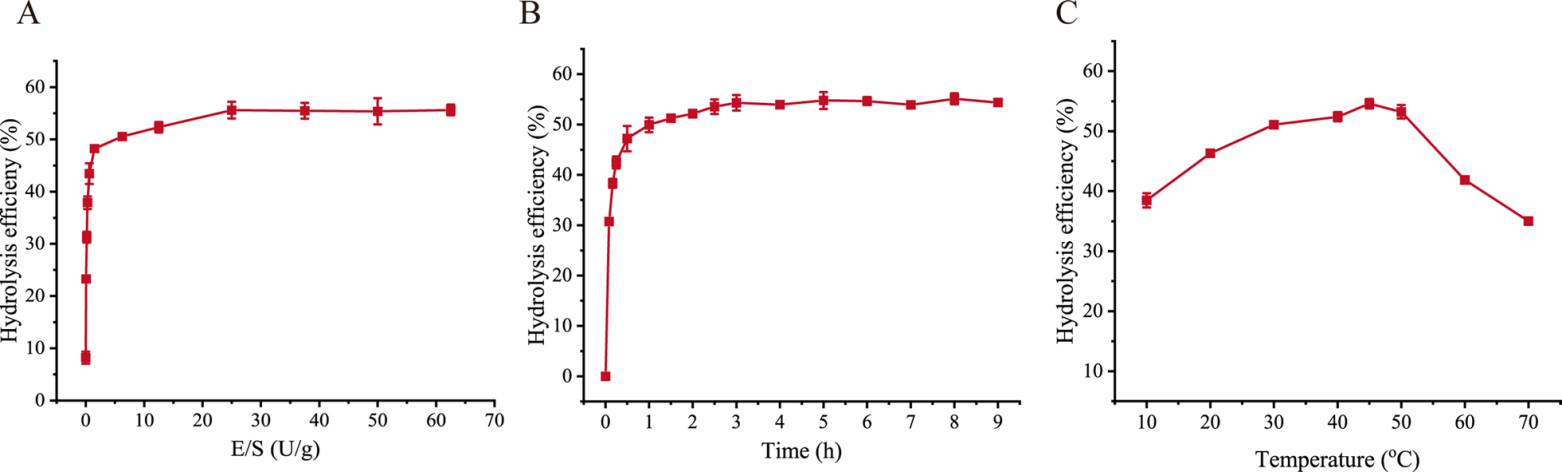
Optimization of the enzymatic hydrolysis parameters of *L. japonica* roots by the fermentation broth supernatant (FBS) of strain A3. **A** effect of E/S ratio on the hydrolysis efficiency. *L. japonica* roots were hydrolyzed by the FBS with different E/S ratios (0, 0.05, 0.15, 0.3125, 0.625, 6.25, 12.5, 25, 37.5, 50 or 62.5 U/g) at 45 °C. **B** effect of hydrolysis time on the hydrolysis efficiency. *L. japonica* roots were hydrolyzed by the FBS from 5 min to 9 h with an E/S ratio of 25 U/g. **C** effect of hydrolysis temperature on the hydrolysis efficiency. Hydrolysis of the FBS on *L. japonica* roots was performed at different temperatures (10, 20, 30, 40, 45, 50, 60 or 70 °C) with an E/S ratio of 25 U/g for 3 h

To investigate the optimal hydrolysis conditions on *L. japonica* roots of the FBS, 3 enzymatic hydrolysis parameters, enzyme-substrate ratio (E/S ratio), hydrolysis time and hydrolysis temperature, were optimized. The hydrolysis efficiency reached the maximum when the E/S ratio was ≧ 25 U/g and the hydrolysis time was ≧ 3 h (Fig. 5A, B). The optimal temperature for the hydrolysis of *L. japonica* root powder by the FBS of strain A3 was 45 °C (Fig. 5C). Under the optimal conditions for hydrolysis (25 U/g for the E/S ratio, 3 h for hydrolysis time and 45 °C for hydrolysis temperature), the hydrolysis efficiency of *L. japonica* root powder reached 54.58 ± 0.79%, indicating that the *L. japonica* roots were efficiently degraded by the FBS of strain A3.

**Fig. 6:**
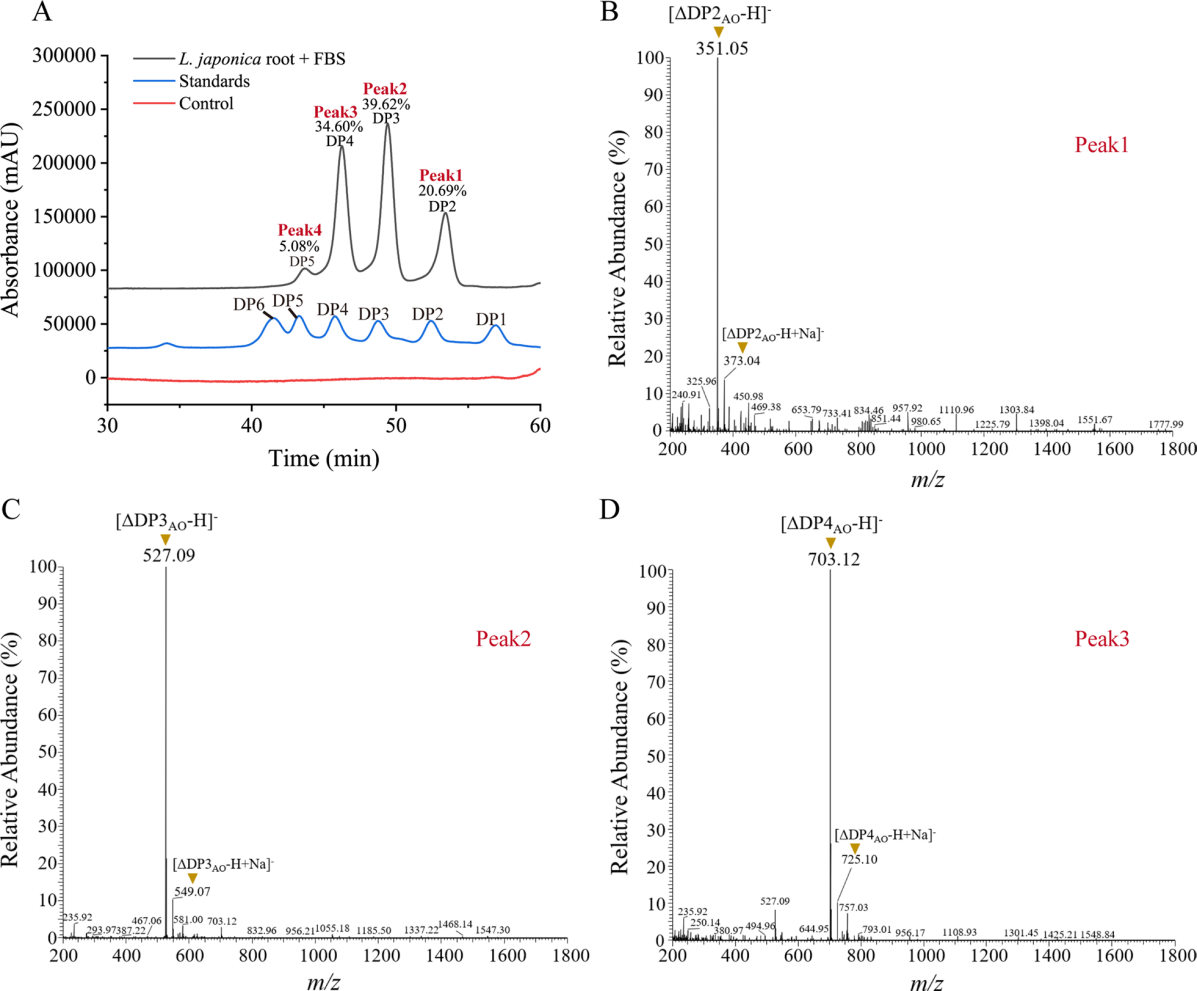
Analyses of the degradation products of *L*. *japonica* roots by the fermentation broth supernatant (FBS) of strain A3. **A** HPLC analysis of the degradation products. *L. japonica* roots treated with the pre-inactivated FBS was taken as the control. Mannuronate oligomers from DP1 to DP6 were taken as the standards. The percentage numbers indicate the percentage of the AO area to the total chromatograph area of the AOs. **B–D** LTQ-Orbitrap-MS analysis of the major peaks in (**A**) in the negative-ion mode. *DP* degree of polymerization, *AO* alginate oligosaccharide

To characterize the hydrolysate generated from the hydrolysis of *L. japonica* roots by the FBS, the composition of the hydrolysate was analyzed by high performance liquid chromatography (HPLC) and LTQ-Orbitrap-MS. HPLC result indicated that the hydrolysate consisted of 4 components (Fig. 6A). Based on subsequent LTQ-Orbitrap-MS analysis, the 3 main components were identified as alginate dimers, trimers and tetramers (Fig. 6B–D). Another product, which was too small to be collected for a further LTQ-Orbitrap-MS analysis, corresponded to alginate pentamers based on the alginate oligomer standards (Fig. 6A). Among the products, alginate trimers and tetramers were the most abundant, accounting for 39.62% and 34.60% of the product amount, respectively, followed by alginate dimers (20.69%) and alginate pentamers (5.08%) (Fig. 6A).

Till now, only 2 studies have reported the AOs preparation directly from brown algae by fermentation of microbial strains [24, 25]. The engineered *Yarrowia lipolytica* strain with an alginate lyase gene was reported to produce AOs with DP2 and DP3 after 72 h-fermentation in the *L. japonica* liquid medium pretreated by enzymes [24]. An alginate lyase-producing *Bacillus litoralis* strain can degrade *Sargassum horneri* water extract pretreated by autoclave to produce AOs mainly at DP2 after 12 h-fermentation [25]. In our study, *L. japonica* roots were used in AOs preparation. Compared with *L. japonica*, the cost of using *L. japonica* roots as the raw material is lower, and no pretreatment except for milling is necessary, saving energy. In addition, the preparation of AOs by the FBS of strain A3 is time-saving, requiring only 3 h. AOs with different DPs have been reported to have distinct bioactivities [36]. AOs produced from the *L. japonica* roots by the FBS of strain A3 are different from those produced in the reports above, indicating that they may have different functions. Especially, the AOs produced by the FBS of strain A3 may be applied in agriculture, as the bioactivities of AOs at DP2-4 have been extensively investigated in plants [14–16]. In addition, the advantages of the low fermentation cost and short time for AOs preparation by strain A3 are also beneficial for the large-scale agricultural usage of the prepared AOs.

### The efficiency of the process for AOs preparation from *L. japonica* roots with the FBS on the laboratory scale

**Fig. 7:**
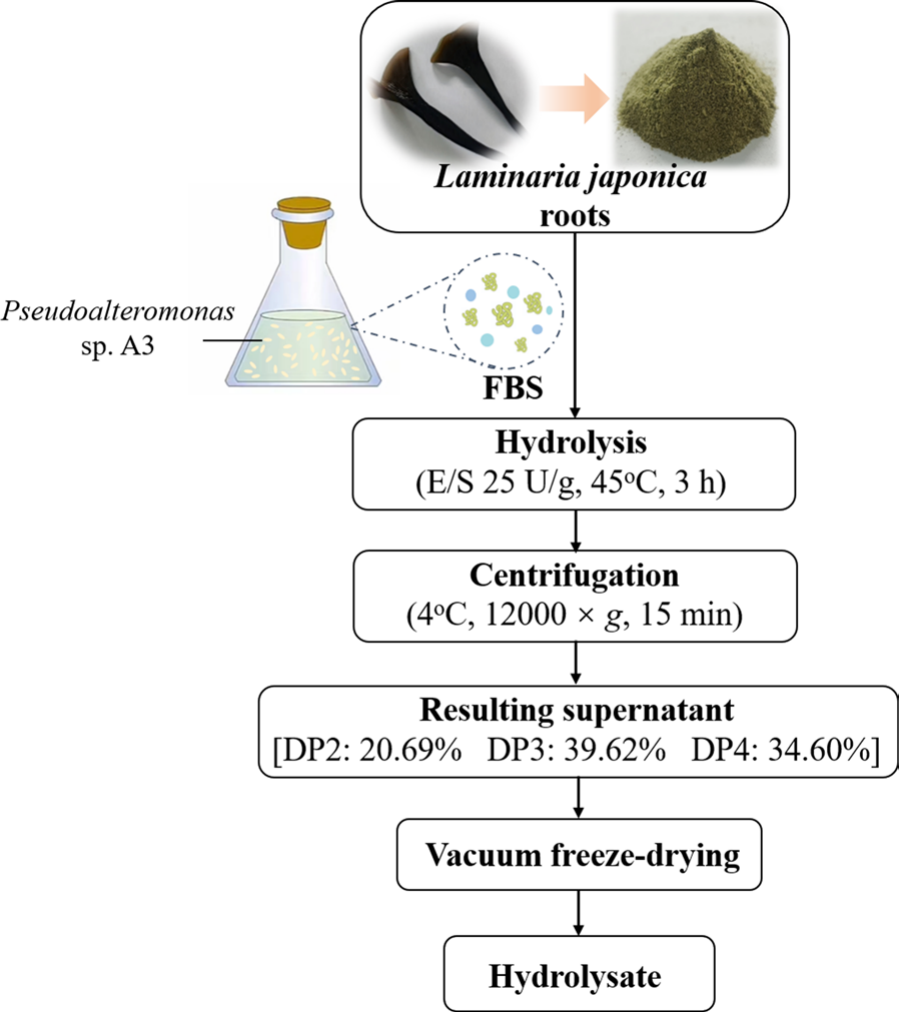
Flow sheet for AOs preparation from *L*. *japonica* roots with the fermentation broth supernatant (FBS) of strain A3

Based on the above results, a process for AOs preparation from *L. japonica* roots with the FBS of strain A3 on the laboratory scale were established (Fig. 7). In the process, the hydrolysis efficiency of *L. japonica* roots reached 54.58 ± 0.79%, as described above. The AOs production, the total sugar production and the AOs purity were then determined to characterize the *L*. *japonica* root hydrolysate obtained by the established process.

On account of the vast majority of uronic acids present in alginate in *L. japonica* [24], the AOs production was measured with the sulfamate/m-hydroxydiphenyl method, a common method for the determination of uronic acids [37]. The AOs production was determined to be 331.08 ± 8.20 mg/g *L*. *japonica* roots (Table 5), accounting for 80.36% of the total amount (41.20%) of alginate in the *L*. *japonica* roots, which indicated that the majority of alginate in *L*. *japonica* roots was degraded by the FBS in the process. Compared with the AOs production (131 mg/g *L. japonica*) of the alginate lyase AlgL7 on *L. japonica* [26], the AOs production of the FBS of strain A3 on *L. japonica* roots was much higher. The total sugar production was 389.36 ± 20.20 mg/g *L*. *japonica* roots. Thus, the purity of the prepared AOs, referring to the proportion of the AOs production to the total sugar production, was 85.03% (Table 5). These results showed that substantial AOs with considerable AOs purity were produced by the process. Therefore, the process is feasible for AOs production directly from *L. japonica* roots.

**Table 5:** Characterization of the hydrolysate of the *L*. *japonica* roots

Treatment	Total sugar production (mg/g)	AOs production (mg/g)	AOs distribution (DP)	AOs purity (%)
Fermentation broth supernatant of strain A3	389.36 ± 20.20	331.08 ± 8.20	Mainly 2–4	85.03

*DP* degree of polymerization

## Conclusion

Herein, we screened a strain *Pseudoalteromonas agarivorans* A3 that can efficiently decompose *L. japonica* to generate AOs mainly relying on its secreted alginate lyases. By optimizing the alginate lyase production of the FBS and the hydrolysis parameters, both of which used the underutilized *L. japonica* roots in the food industry as raw material, we established an efficient, cost-effective and green process for AOs production. In this process, *L. japonica* roots were hydrolyzed efficiently and substantial AOs mainly at DP2-4 with considerable purity were obtained. The developed process is a practical process for AOs production directly from *L. japonica* roots with strain A3, which may have a promising application potential in industry and agriculture. This represents the first study on AOs production from direct degradation of *L. japonica* roots, which converts the leftover material into a high-value compound.

## Materials and methods

### Materials

The sun-dried *L. japonica*, including the roots and the blades, was obtained from the local seafood market (36.08° N, 120.35° E), which was first soaked and washed with water to remove dirt and salt. The washed *L. japonica* roots were then dried and milled into powder. The *L. japonica* root powder was passed through an 80-mesh sieve, and stored at room temperature until use. The content of alginate in the dried *L. japonica* blade and *L. japonica* root used in this study was determined to be 44.40% and 41.20%, respectively, according to the previously reported extraction method of alginate [38]. Sodium alginate was purchased from Sinopharm Co., Ltd (China). AO standards (purity ≧ 97%) were purchased from Zzstandard (China). Glucose, D-glucuronic acid and sea salt were purchased from Sigma (USA). The TEM 200 copper grid was purchased from Beijing Zhongjingkeyi Technology Co., Ltd (China).

### Screening of *L. japonica*-decomposing bacteria

The fresh *L. japonica* sample was collected from a kelp farm in Weihai, China and stored in a sterile collection bag in ice for 3 days before being further processed. The sample (20 g) was cut into small pieces (1 cm × 1 cm) and put in 200 ml sterile seawater containing 3% (w/v) sea salts in a 500 ml flask, which was shaken at 180 rpm and 25 °C for 15 min. The resultant solution was serially diluted to 10^−4^ to 10^−6^ dilution and then each diluted solution was spread on the screening plates containing a medium composed of 0.5% (w/v) sodium alginate, 3% sea salts and 1.5% (w/v) agar [39]. The plates were incubated at 25 °C.

After incubation at 25 °C for 3–5 days, morphologically distinct colonies emerged on the plates, which were then separated by repeatedly streaking on the 2216E marine agar plates to obtain the purified isolates. The obtained isolates were re-streaked on the screening plates, incubated at 25 °C for 5 days and stained by Lugol’s solution [40]. The HD/CD ratio was used as the standard for a preliminary evaluation of the extracellular alginolytic activity of the isolates. Isolates with a HD/CD ratio ≧ 3 were then inoculated into the liquid medium containing 0.5% sodium alginate and 3% sea salt, and cultured at 25 °C for 24 h to further confirm the alginate-degrading ability. The screened alginate-degrading strains were inoculated into the 4 ml medium containing 3% sea salts and a piece of *L. japonica* blade (1 cm × 1 cm) and cultured at 180 rpm and 25 °C. The decomposition of *L. japonica* was observed at 0, 6, 12 and 24 h. Strain A3 showing the highest *L. japonica-*decomposing ability was screened for further study.

### TEM analysis

Strain A3 was cultured in 2216E marine broth at 25 °C, 180 rpm for 12 h. The cells were collected and washed three times with sterile seawater. The cells were then transferred to a TEM 200 copper grid and incubated for 2 min. The grid was dried, negatively stained with 3% (w/v) aqueous uranyl acetate for 1.5 min. After air drying, the cells were observed and photographed using a Tecnai G2 F20 microscope (USA, FEI).

### Alginate lyase assay

The alginolytic activity of the FBS was measured by the dinitrosalicylic acid (DNS) method [41] with glucose as the standard. A 200-µl reaction mixture containing 2 mg/ml sodium alginate, 0.5 M NaCl and 20 µl FBS in 50 mM Tris-HCl (pH 8.0) was incubated at 45 °C for 15 min. Then, the reaction was terminated after the addition of 100 µl DNS. The reaction mixture was boiled for 10 min for color development. After that, the absorbance of the mixture at 540 nm was measured. One unit (U) of enzyme activity was defined as the amount of enzyme required to release 1 µmol reducing sugars per min. The reaction system containing the pre-inactivated FBS under the same conditions was taken as the control.

Alginolytic activity of the optimized FBS was also determined by the UA method [42]. The reaction mixture containing 50 mM Tris-HCl (pH 8.0), 2 mg/ml sodium alginate, 0.5 M NaCl and 20 µl FBS was incubated at 45 °C for 15 min. The reaction was then terminated by boiling the mixture for 10 min. The increase in the absorbance at 235 nm (A_235_) resulted from the release of unsaturated uronic in the mixture was monitored. One unit (U) of enzyme activity was defined as the amount of enzyme required to cause an increase of 0.1 at 235 nm per minute.

### Optimization of the fermentation conditions of strain A3 for alginate lyase production

#### Optimization with single factor method

To determine the optimal fermentation temperature and time for alginate lyase production, strain A3 was cultured for different times (0, 3, 6, 9, 12, 24, 36, 48, 60 or 72 h) at 10, 15, 20, 25 or 30 °C, 180 rpm in 50 ml basic fermentation medium containing 1% (w/v) *L. japonica* root powder and 3% sea salts. To optimize the content of *L. japonica* root powder in the fermentation medium, strain A3 was inoculated into the medium containing 3% sea salts and different concentrations of *L. japonica* root powder (0.1, 0.25, 0.5, 0.75, 1 or 1.25%) and cultured at 20 °C, 180 rpm for 24 h. To determine the optimal inorganic nitrogen source, an additional 0.13% (w/v) nitrogen source, NH_4_Cl, NH_4_HCO_3_, NH_4_NO_3_ or (NH_4_)_2_SO_4_, was added into the basic fermentation medium. To determine the optimum concentration of NH_4_NO_3_, NH_4_NO_3_ was added into the basic fermentation medium at different final concentrations (0, 0.5, 1.0, 1.5, 2.0, 2.5, 3.0, 3.5 or 4%). Strain A3 was cultured in the media at 20 °C, 180 rpm for 24 h. After fermentation, the alginate lyase activity of the FBS was determined.

#### Optimization with RSM

RSM was further performed based on the single factor trials. The designation of the experiments and the data analysis were conducted by using the Design Expert 12 software.

In the PB design, the first step of RSM, 11 variables, including NH_4_NO_3_ concentration, rotation speed, *L. japonica* roots concentration, fermentation time, fermentation temperature, pH, inoculum size, sea salt concentration and 3 dummy variables, were selected in 12 experiments. Each variable was tested at low (− 1) and high (+ 1) levels (Table 2) [43]. The path of steepest ascent (descent) experiment started from the center point (“0” level) in the PB design and the moving directions of significant variables were related to the regression coefficients obtained by the PB design (Table 3) [44]. The levels where the alginate lyase production peaked were taken as “0” level for the following CCD. CCD with 5 coded levels (“− 2”, “− 1”, “0”, “+ 1”, and “+ 2”) was used for obtaining the true optimal fermentation conditions. A total of 30 experiments were performed with 6 replications at level “0” (Table 4), and the results were analyzed using a second-order equation (Additional file 1: Formula S2).

### Optimization of the hydrolysis parameters of the FBS on *L. japonica* roots

To determine the optimal E/S ratio, 0.16 g *L. japonica* root powder was reacted with the FBS with different E/S ratios (0, 0.05, 0.15, 0.3125, 0.625, 6.25, 12.5, 25, 37.5, 50 or 62.5 U/g) for 12 h. To determine the optimal hydrolysis time, *L. japonica* root powder was hydrolyzed by the FBS from 5 min to 9 h with an E/S ratio of 25 U/g. To determine the optimal hydrolysis temperature, *L. japonica* root powder was hydrolyzed by the FBS at different temperatures (10, 20, 30, 40, 45, 50, 60 or 70^o^C) for 3 h with an E/S ratio of 25 U/g. After hydrolysis, the reaction mixture was centrifuged at 12,000 × *g* for 10 min. After centrifugation, the supernatant was collected as the *L. japonica* root hydrolysate, and the precipitated *L. japonica* root powder was freeze dried and weighed for calculating the hydrolysis efficiency of *L. japonica* root powder by the following equation:

1 $${\text{Hydrolysis efficiency}}\left( \% \right) = \left( {{\text{W}}_{1} {-}{\text{W}}_{2} } \right)/{\text{W}}_{1} \times 100.$$

where W_1_ and W_2_ are the weight of the *L. japonica* root powder samples before and after being hydrolyzed.

### Degradation products analysis

The degradation products of the FBS of strain A3 towards *L. japonica* piece and *L. japonica* roots were identified by high-resolution LTQ-Orbitrap-MS (Thermo, USA) in the negative-ion mode. The source voltage and capillary temperature were set at 3.6 kV and 275 °C, respectively. The mass acquisition range was 160–1500 *m/z* or 200–1500 *m/z*.

The AOs composition in the *L. japonica* root hydrolysate produced by the FBS of strain A3 was analyzed by HPLC on a Superdex Peptide 10/300 GL column (GE Healthcare, USA) [45]. The AOs in the hydrolysate were eluted with 0.2 M NH_4_HCO_3_ at a flow rate of 0.3 ml/min and detected by a UV detector at 210 nm. The commercial saturated mannoronate oligomers were taken as the standards. The proportion of each AO was calculated according to the percentage of the AO area to the total chromatograph area of the AOs [46].

Total sugar content of the degradation products was measured by the phenol–sulfuric acid method [47] using d-glucuronic acid as the standard. The content of uronic acid in the degradation products was analyzed by the sulfamate/*m*-hydroxydiphenyl assay described by Filisetti-Cozzi [37] using d-glucuronic acid as the standard.

### Genome sequencing and analysis

Genomic DNA of strain A3 was extracted with a bacterial DNA kit (Omega, USA) and sequenced on the Illumina platform (Biozeron, China). Genome assembly was performed using ABySS v2.1.5. The genome data of strain A3 was submitted to NCBI (GenBank: JAPCKL000000000.1). The 16S rRNA gene sequence of strain A3 was aligned on the Ezbiocloud database (http://www.ezbiocloud.net). The phylogenetic analysis was performed by MEGA X [48]. The putative CAZymes of strain A3 were predicted by dbCAN meta server [49].

### Secretome analysis

Strain A3 was cultured in the 100 ml medium containing 25 pieces of *L. japonica* blade (~ 0.1 g/ml, 1 cm × 1 cm) and 3% sea salts at 25 °C for 18 h. The supernatant of the culture was collected after centrifugation (10,000×*g*) and proteins in the supernatant were processed and identified according to the method described by Cheng et al. [50]. The secretome data was uploaded to the ProteomeXchange Consortium (http://proteomecentral.proteomexchange.org) via the iProX partner repository [51] under the accession number PXD037880.

### Supplementary Information

**Additional file 1: Table S1.** The analysis of variance (ANOVA) results of the model for alginate lyase production of strain A3 by the Plackett–Burman (PB) design. **Table S2.** The ANOVA results of the model for alginate lyase production of strain A3 by the central composite design (CCD). **Formula S1.** The second-order equation of central composite design (CCD) established by multiple regression analysis. **Formula S2.** The second-order equation used for the analysis of the central composite design (CCD) result.

## Data Availability

All data generated or analyzed during this study are included in this article and the additional file.
