# Supplementary material for: Cost-effective production of alginate oligosaccharides from Laminaria japonica roots by Pseudoalteromonas agarivorans A3
Source: Microb Cell Fact. 2023 Sep 9;22:179. doi: 10.1186/s12934-023-02170-7 (PMC10492272; doi:10.1186/s12934-023-02170-7)
Supplement: Supplementary file 1 — Additional file 1: Table S1. The analysis of variance (ANOVA) results of the model for alginate lyase production of strain A3 by the Plackett–Burman (PB) design. Table S2. The ANOVA results of the model for alginate lyase production of strain A3 by the central composite design (CCD). Formula S1. The second-order equation of central composite design (CCD) established by multiple regression analysis. Formula S2. The second-order equation used for the analysis of the central composite design (CCD) result. [file 12934_2023_2170_MOESM1_ESM.pdf]

Table S1. The analysis of variance (ANOVA) results of the model for alginate lyase production of strain A3 by the Plackett-Burman (PB) design.

Variables[*]	Sum of squares	Coefficient estimate	F-value	p-value Prob > F
Model	31.80	-	33.55	0.0075[a]
Intercept	-	2.93	-	-
X_1	1.76	0.3833	14.88	0.0308[a]
X_2	0.0027	-0.0150	0.0228	0.8896
X_3	1.09	0.3017	9.22	0.0561
X_4	0.6912	0.2400	5.83	0.0946
X_5	21.60	-1.34	182.30	0.0009[a]
X_6	4.47	0.6100	37.68	0.0087[a]
X_7	1.78	0.3850	15.01	0.0304[a]
X_8	0.4017	0.1850	3.47	0.1596

[*] X_1, NH_4NO_3 (w/v, %); X_2, rotation speed (rpm); X_3, *L. japonica* root powder (w/v, %); X_4, fermentation time (h); X_5, fermentation temperature (°C); X_6, pH; X_7, inoculum size (%); X_8, sea salt concentration (w/v, %).

[a] Model terms are significant.

Table S2. The ANOVA results of the model for alginate lyase production of strain A3 by the central composite design (CCD).

Source*	df	Sun of squares	Mean square	*F*-value	*p*-value
Model	14	23.84	1.70	21.57	< 0.0001[a]
X_1	1	1.39	1.39	17.57	0.0008[a]
X_2	1	1.59	1.59	20.09	0.0004[a]
X_3	1	1.98	1.98	25.06	0.0002[a]
X_4	1	3.50	3.50	44.38	< 0.0001[a]
$X_1 X_2$	1	0.0390	0.0390	0.4941	0.4929
$X_1 X_3$	1	0.0218	0.0218	0.2756	0.6073
$X_1 X_4$	1	0.2627	0.2627	3.33	0.0881
$X_2 X_3$	1	0.2627	0.2627	3.33	0.0881
$X_2 X_4$	1	0.9555	0.9555	12.10	0.0034[a]
$X_3 X_4$	1	0.0233	0.0233	0.2946	0.5953
X_1^2	1	6.68	6.68	84.60	< 0.0001[a]
X_2^2	1	1.78	1.78	22.54	0.0003[a]
X_3^2	1	8.44	8.44	106.90	< 0.0001[a]
X_4^2	1	0.1479	0.1479	1.87	0.1912
Residual	15	1.18	0.0789		
Lack of Fit	10	0.8974	0.0897	1.56	0.3244
Pure Error	5	0.2868	0.0574		
Cor Total	19	25.03			

* X_1, NH_4NO_3 (w/v, %); X_2, fermentation temperature (ºC); X_3, pH; X_4, inoculum size (%).

[a] Model terms are significant.

Formula S1. The second-order equation of central composite design (CCD) established by multiple regression analysis.

$$Y\ (\text{U/ml}) = 8.21 + 0.2404\ X_1 - 0.2571\ X_2 + 0.2871\ X_3 + 0.3821\ X_4 + 0.0494\ X_1 X_2 - 0.0369\ X_1 X_3 - 0.1281\ X_1 X_4 + 0.1281\ X_2 X_3 + 0.2444\ X_2 X_4 + 0.0381\ X_3 X_4 - 0.4934\ X_1^2 - 0.2547\ X_2^2 - 0.5547\ X_3^2 - 0.0734\ X_4^2$$

where Y is the alginate lyase production of the fermentation broth supernatant (FBS) of strain A3, X_1 the concentration of NH_4NO_3, X_2 the fermentation temperature, X_3 the pH, and X_4 the inoculum size.

Formula S2. The second-order equation used for the analysis of the central composite design (CCD) result.

$$Y = \beta_0 + \sum \beta_i x_i + \sum \beta_{ii} x_i^2 + \sum \beta_{ij} x_i x_j$$

where Y is the predicted response, β_0 the intercept term, β_i the linear coefficients, β_{ii} the quadratic coefficients, β_{ij} the interactive coefficients, and x_i and x_j the coded independent variables [1].

Reference

1. Li X, Xu T, Ma X, Guo K, Kai L, Zhao Y, Jia X, Ma Y. Optimization of culture conditions for production of *cis*-epoxysuccinic acid hydrolase using response surface methodology. Bioresour Technol 2008;99(13):5391-5396. https://doi.org/10.1016/j.biortech.2007.11.017.
